# Highly Contiguous Nanopore Genome Assembly of Chlamydomonas reinhardtii CC-1690

**DOI:** 10.1128/MRA.00726-20

**Published:** 2020-09-10

**Authors:** Samuel O’Donnell, Frederic Chaux, Gilles Fischer

**Affiliations:** a Sorbonne Université, CNRS, Institut de Biologie Paris-Seine, Laboratory of Computational and Quantitative Biology, Paris, France; Vanderbilt University

## Abstract

The current Chlamydomonas reinhardtii reference genome remains fragmented due to gaps stemming from large repetitive regions. To overcome the vast majority of these gaps, publicly available Oxford Nanopore Technology data were used to create a new reference-quality *de novo* genome assembly containing only 21 contigs, 30/34 telomeric ends, and a genome size of 111 Mb.

## ANNOUNCEMENT

The model species Chlamydomonas reinhardtii is important for our understanding of the structure and function of both chloroplasts and cilia. However, we are inhibited by the lack of contiguity of the current reference nuclear genome assembly, which contains 1,495 contigs representing 17 chromosomes and 37 minor scaffolds (1) (Table 1). In contrast to other eukaryote models, the C. reinhardtii genome is GC rich (64%) and not compact; chromosomes are up to 9 Mb long, genes carry on average 7 introns of >350 bp (1), and transposable elements are believed to be relatively active (2). Large regions of repetitive material unable to be spanned by previous sequencing technologies also affect the genome contiguity. In order to improve on this, recently released Oxford Nanopore Technology sequencing data, exploited solely for the detection of epigenetic markers (3), were used for *de novo* assembly of the nuclear genome. This new assembly is for the strain CC-1690 mt+ (“21 gr”) (3), which is used in numerous laboratories and is genetically distant by 0.08% from CC-503 mt+ (4), which has only been used to generate the reference genome (1).

**TABLE 1:** Genome assembly statistics for the CC-503 reference, raw CC-1690 assemblies, and the final CC-1690 assembly

Assembly	No. of contigs	Genome size (Mb)	*N*_50_ (Mb)	*L*_50_	*N*_90_ (Mb)	*L*_90_	GC content (%)
CC-503 v5	1,495	107.050	0.215	141	0.039	596	64.08
CC-1690 raw avg^*a*^	62.2	114.169	3.739	11.2	1.240	31.2	64.03
CC-1690 final	21	111.112	6.886	7	4.015	15	64.13

a Mean values were determined from 5 raw *de novo* genome assemblies.

Initially, raw fast5 files (3) were re-base called using Guppy v3.4.5, adapters were removed using Porechop v0.2.3 (https://github.com/rrwick/Porechop), and FASTQ files were downsampled to various depths of coverage (40×, 50×, and 60×) using Filtlong v0.2 (https://github.com/rrwick/Filtlong) and applying the parameter “--length_weight 10.” For all tools, default parameters were used except where otherwise noted. The read subset with 40× coverage contained 87,192 reads with both a mean read length and *N*_50_ value of 55 kb. In total, 5 raw genome assemblies were first achieved using both SMARTdenovo v1 (https://github.com/ruanjue/smartdenovo) and Canu v2 (5) with the 3 coverage depths and then polished by 3 and 2 rounds of Racon (6) and Medaka (https://github.com/nanoporetech/medaka), respectively. These long-read polished assemblies had an average of 62 contigs and an average size of 114 Mb. Following this, each assembly’s contigs were evaluated based on 2 primary stats, their contiguity against the current C. reinhardtii genome v5.6 (1) and the presence of telomeric repeats at their ends. Contigs for the final assembly were then manually chosen from any of the initial assemblies in order to reduce the total number of contigs necessary to cover the entire reference nuclear genome and maximize the number of telomeric ends. Additionally, to further improve contiguity, 11 overlapping contigs were manually joined after validating the structure with both another contig and multiple long reads (more than 3 reads in all cases). Next, publicly available Illumina data, from the same strain, were downloaded (SRA number SRR1734612) (7) and used to enhance the further assembly accuracy using 3 rounds of Pilon v1.22 (8). In the end, this generated an assembly containing 21 contigs, 111 Mb, and 30/34 ends with telomeric repeats. Finally, a 50-N scaffold, spanning an unassembled region, was placed in chromosomes 4, 12, and 13 based on the structure of the reference genome, and one large contig was left unplaced.

A BUSCO v4.0.6 analysis (9) was performed, using the genome mode, alongside the current C. reinhardtii reference (1). The new assembly contained 5 more complete benchmarking universal single-copy orthologs (BUSCOs) than the reference, totaling 1,515/1,519 (99.7%) (BUSCO data set chlorophyta_odb10).

### Data availability.

All of the raw fast5 files are available under the ENA accession number PRJEB31789. The genome sequence is available under the GenBank accession number JABWPN000000000.
